# Antioxidant Peptides and Protein Hydrolysates from Tilapia: Cellular and In Vivo Evidences for Human Health Benefits

**DOI:** 10.3390/foods13182945

**Published:** 2024-09-18

**Authors:** Wen-Jie Ng, Fai-Chu Wong, Fazilah Abd Manan, Yit-Lai Chow, Ai-Lin Ooi, Mei-Kying Ong, Xuewu Zhang, Tsun-Thai Chai

**Affiliations:** 1 Department of Allied Health Sciences, Faculty of Science, Universiti Tunku Abdul Rahman, Kampar 31900, Malaysia; ngwj@utar.edu.my; 2 Centre for Biomedical and Nutrition Research, Universiti Tunku Abdul Rahman, Kampar 31900, Malaysia; 3 Department of Chemical Science, Faculty of Science, Universiti Tunku Abdul Rahman, Kampar 31900, Malaysia; wongfc@utar.edu.my; 4 Center for Agriculture and Food Research, Universiti Tunku Abdul Rahman, Kampar 31900, Malaysia; chowyl@utar.edu.my (Y.-L.C.); ooial@utar.edu.my (A.-L.O.); ongmk@utar.edu.my (M.-K.O.); 5 Department of Biosciences, Faculty of Science, Universiti Teknologi Malaysia, Skudai 81310, Malaysia; m-fazilah@utm.my; 6 Department of Biological Science, Faculty of Science, Universiti Tunku Abdul Rahman, Kampar 31900, Malaysia; 7 Department of Agricultural and Food Science, Faculty of Science, Universiti Tunku Abdul Rahman, Jalan Universiti, Bandar Barat, Kampar 31900, Malaysia; 8 College of Food Science and Engineering, South China University of Technology, 381 Wushan Road, Guangzhou 510640, China; snow_dance@sina.com; 9 Era Biotechnology (Shenzhen) Co., Ltd., Shenzhen 518115, China

**Keywords:** antiaging, antifatigue, anti-inflammatory, aquaculture, bioactive compounds, enzymatic hydrolysis, functional food, oxidative stress, wound healing

## Abstract

Antioxidant peptides derived from aquatic organisms have attracted tremendous research interest due to their potential applications in human health. Tilapia is one of the most widely farmed aquaculture species globally. The current understanding of tilapia-derived antioxidant peptides is gradually expanding. This review discusses the current knowledge of peptides and protein hydrolysates derived from tilapia muscle, skin, and scales, whose antioxidant capacity has been validated in various cellular and in vivo models. To date, at least 16 peptides and several hydrolysates have been identified from tilapia that protect human and non-human cell models against oxidative injury. Tilapia hydrolysates and peptide mixtures have also shown protective effects in animal models of oxidative stress-associated diseases and exercise-induced oxidative injury and fatigue. The key mechanisms of tilapia hydrolysates and peptide mixtures involve enhancing antioxidant enzyme activities and suppressing radical production. Notably, such hydrolysates also exerted additional in vivo functions, such as anti-inflammatory, anti-diabetic, wound healing, and antiaging properties. Taken together, tilapia-derived antioxidant peptides and hydrolysates represent a valuable source of functional ingredients for applications in functional food, dietary supplements, and therapeutic applications. Continued research into their health benefits is warranted in the future.

## 1. Introduction

Food-derived antioxidant peptides, often comprising between 2 and 20 amino acids, can exert a diversity of antioxidant action either in vitro or in vivo. Their modes of action could involve (i) direct scavenging of free radicals, such as reactive oxygen species (ROS) and reactive nitrogen species (RNS), (ii) direct interception of free radical chain reactions, or (iii) regulation of the gene and protein expression of oxidative and antioxidant enzymes [1,2,3]. Current interest in antioxidant peptides is propelled by their potential applications in the development of functional foods and peptide-based therapeutic agents. Furthermore, the potential application of antioxidant peptides as food-derived preservatives or additives has also been highlighted [1,2,4].

The past two decades have seen a surge in research on the discovery of antioxidant peptides originating from foods and food-processing by-products. Various plant- and animal-derived food materials have been demonstrated to be rich sources of antioxidant peptides [1,5,6]. Numerous studies have adopted the enzymatic hydrolysis approach to liberate encrypted antioxidant peptide fragments from food proteins. To this end, commercially available proteases, such as Alcalase^®^, Neutrase^®^, papain, and trypsin, have been employed. Moreover, microbial fermentation has also been used to generate antioxidant peptides from food proteins [1,7]. Fish and fishery by-products, among various animal protein-enriched raw materials, have continuously attracted the attention of bioactive peptide researchers worldwide [6,8,9]. For instance, research has identified antioxidant peptides from the muscle tissues of various fish species, including mackerel [10], silver carp [11], and monkfish [12]. Furthermore, antioxidant peptides have been identified from fishery by-products such as sturgeon ovaries [13], monkfish swim bladders [14], skipjack tuna roe [15], Alaska pollock skin collagen [16], horse mackerel viscera [17], and redlip croaker scales [18].

Tilapia, farmed in 126 countries, is one of the most valuable aquaculture species, with a global production of 7 million tons, contributing to a global market value of USD 12 billion in 2020 [19,20,21]. At present, there is a growing body of cellular and in vivo evidence substantiating the value of tilapia as a source of biologically relevant antioxidant peptides. The potential applications of tilapia-derived antioxidant peptides in promoting human health and attenuating oxidative stress-mediated pathological conditions have been proposed by an increasing number of studies [22,23,24]. Further enhancing their appeal, the majority of tilapia are farm-raised and not classified as an endangered species; this makes tilapia peptides a favorable choice from a wildlife preservation perspective. From a socio-economic standpoint, tilapia is an inexpensive and cost-competitive fish species, well-recognized for its fast growth rate, ability to grow in poor water quality, and high disease resistance [25]. These factors contribute to the sustainability and economic viability of utilizing tilapia for peptide production.

This review explores the current understanding of how tilapia-derived protein hydrolysates and peptides protect cells and animals against oxidative stress. Numerous studies reported the antioxidant activity of tilapia-derived protein hydrolysates and peptides that was demonstrated solely through in vitro chemical assays such as the 2,2-diphenyl-1-picrylhydrazyl and 2,2′-azino-bis(3-ethylbenzothiazoline-6-sulfonic acid) (ABTS) radical scavenging assays [26,27,28]. The evidence of such chemical assays may not accurately reflect the biological relevance of a peptide or any other bioactive compound [29,30]. Thus, in this review, our emphasis is on tilapia-derived antioxidant peptides and protein hydrolysates whose efficacy has been confirmed using at least a cellular or an animal model. This review encompasses relevant studies published in English, which we identified through electronic searches of the Scopus database. We searched the “Article title, Abstract, Keywords” fields using the following search words: “tilapia”, “*Oreochromis*”, “antioxidant”, “antioxidative”, “peptide”, and “hydrolysate”. After carefully screening the abstracts of 319 research articles, we identified 30 studies reporting cellular and in vivo antioxidant effects of tilapia-derived protein hydrolysates and peptides. Review articles were excluded to prioritize primary research.

In this review, we focus on recent progress in the discovery of antioxidant peptides from both edible tissues (e.g., muscles) and non-edible tissues (e.g., scales) of tilapia. The latter is of significance, considering that the valorization of fish processing by-products, including those from tilapia, is currently an important research direction worldwide [9]. In the tilapia fillet industry, for instance, the fillet typically represents 33% of the fish, with a large portion (67%) of the total weight consisting of processing by-products, including the head, carcass, viscera, fins, skin, and scales [31]. In addition to their biological effects and underlying mechanisms, the structure–activity relationship of tilapia-derived antioxidant peptides will be discussed. The potential applications of the hydrolysates and peptides in the development of functional foods and therapy will be explored. Future research directions will also be presented. This review prioritizes studies that have successfully identified the sequences of tilapia-derived antioxidant peptides. Nevertheless, protein hydrolysates are raw materials for the future discovery of antioxidant peptides. Protein hydrolysates may also be more cost-effective than pure peptides in the development of nutraceutical and functional food ingredients [32]. Notably, current in vivo evidence on the effects of tilapia-derived peptides comes predominantly from experiments conducted using protein hydrolysates rather than specific peptides. Thus, the antioxidant potency of tilapia-derived peptide fractions or hydrolysates will also be discussed where appropriate. By comprehensively analyzing the current state of knowledge, this review will provide insights into the potential of tilapia-derived antioxidant peptides in human health promotion.

## 2. Tilapia

**Figure 1:**
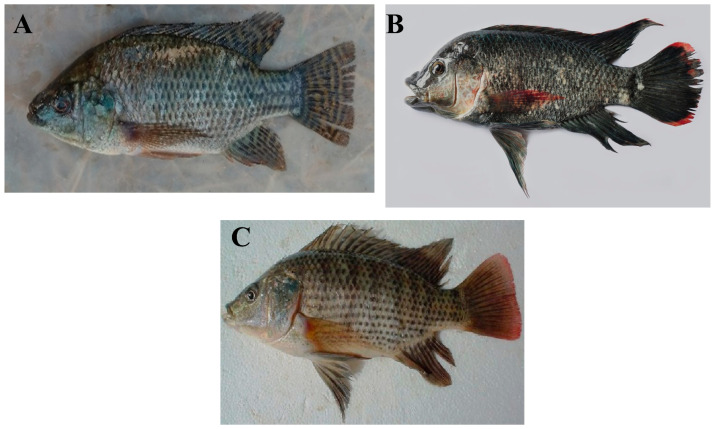
(**A**) Nile tilapia (*Oreochromis niloticus*), (**B**) Mozambique tilapia (*O. mossambicus*), and (**C**) blue tilapia (*O. aureus*). (Photo credits: (**A**)—Cyrus Rumisha, FishBase [36]; (**B**)—Balaram Mahalder, FishBase [36]; (**C**)—Magdy A. Saleh, FishBase [36]).

Tilapia is a freshwater fish of the cichlid family that is native to Africa. Several commercially important species exist, such as the Nile tilapia (*Oreochromis niloticus*), Mozambique tilapia (*O. mossambicus*), and blue tilapia (*O. aureus*) (Figure 1) [33,34,35]. Tilapia is an omnivorous fish that is easy to feed and culture. It can reproduce year-round and can be cultured in fresh, brackish, and seawater. Due to all these characteristics, tilapias have been introduced into many tropical and sub-tropical countries around the world since the 1960s [35].

Tilapia production has quadrupled over the past two decades because of the fish’s suitability for aquaculture, marketability, and stable market prices. In 2020, China was the world’s largest producer of tilapia, with a production of 1.66 million tons, followed by Indonesia (1.23 million tons), Egypt (0.95 million tons), and Brazil (0.34 million tons) [19]. Among the tilapia species, Nile tilapia is rapidly gaining status as a farmed commodity, accounting for 9% of the global inland aquaculture production in 2020. It ranks second after carps (grass carp and silver carp), with a total production volume of 4.407 million tons (Table 1) [37].

In 2019, the global per capita consumption of fish was reported as 20.5 kg, where nearly 40% came from freshwater species (FAO, 2022). In some Asian countries, such as Vietnam and Thailand, along with the increase in domestic consumption of tilapia, the production and export of frozen whole tilapia to the United States doubled in 2022 [38]. The growing global demand for tilapia in recent years can be attributed to consumers’ awareness of the nutritional advantages of aquatic foods, especially fish. Another important factor is the low market price of tilapia compared to other fishes. Other desirable characteristics of tilapia are their firm texture, mild flavor, and availability in various forms, including live, whole fresh, filleted, frozen, smoked, and sashimi [39,40]. Texture is one of the important quality attributes of fish muscle, whether raw or cooked. It is used in the fresh and processed fish industry to assess product quality and consumer acceptability. A firm texture is preferred as the fish flesh will not disintegrate easily [37]. The high nutritional value of tilapia lies in their high protein content (16–25%) and low fat content (0.5–3.0%), in addition to a richness in several essential amino acids and several essential fatty acids such as docosahexaenoic acid and arachidonic acid. Tilapia is also a good source of several micronutrients such as vitamin D and phosphorus [40].

## 3. Production of Antioxidant Hydrolysates and Peptides from Tilapia

Protein hydrolysates and peptides that have been shown to exert antioxidant activities in cellular and animal models have been isolated from tilapia muscle, skin, scales, and viscera (Table 2). Most of these studies began with the production of antioxidant peptides from tilapia by means of hydrolysis with commercial proteases (Table 2). Alcalase^®^, either used alone or in combination with other proteases, was frequently used to produce hydrolysates from tilapia. The preference for Alcalase^®^ may be due to the fact that the protease displays both exo- and endo-protease activities, which allows for a broader specificity of hydrolysis sites, thus providing relatively extensive proteolysis [41]. Some studies have used multiple proteases to generate tilapia protein hydrolysates and peptides, either by in vitro simulation of gastrointestinal (GI) digestion with a combination of different GI proteases such as pepsin and pancreatin [42], sequential hydrolysis with different proteases such as Multifect neutral^®^ followed by Properase E^®^ [43], or a mixture of enzymes such as neutral protease and papain [23] (Table 2). Interestingly, some studies conducting single-enzyme hydrolysis observed a relatively high hydrolysis degree [29,44,45] when compared with those performing multi-enzyme hydrolysis [43,46]. Among the studies summarized in Table 2, the duration of proteolysis ranged from 30 min to 16 h. The higher yield of low molecular weight peptides in the resultant hydrolysate may, at least in part, be attributed to the duration of hydrolysis [41]. When tilapia dorsal meat samples hydrolyzed for 2 to 16 h were compared, the increase in the degree of hydrolysis from 20 to 35% was associated with an increase in the proportion of short peptides (below 330 Da) from 10 to 23% [47].

The isolation of antioxidant peptides from tilapia protein hydrolysates was often accomplished by fractionating the hydrolysates using chromatographic techniques. As presented in Table 3, tilapia protein hydrolysates were initially fractionated by molecular size using gel filtration chromatography with Sephadex G-25 and G-15 resins [42,43,46,48]. The semi-purified peptide fractions were then further purified using reversed-phase high-performance liquid chromatography (RP-HPLC) [42,43,48,53,54]. Finally, the peptides present in the purified RP-HPLC fraction exhibiting the strongest antioxidant activities were identified by means of tandem mass spectrometry, either electrospray ionization–quadrupole time-of-flight mass spectrometry [43,54] or liquid chromatography coupled with tandem mass spectrometry [29,48].

## 4. Cellular Effects

Various in vitro cell lines have been employed to investigate the antioxidative efficacy of tilapia-derived hydrolysates and peptides, focusing on their ability to inhibit ROS generation, prevent oxidative damage, and promote the expression of genes encoding antioxidant enzymes. Non-human cells used in such research included mouse macrophages [48,53], murine microglial cells [54], mouse embryonic fibroblasts [42], porcine jejunal cells [55], and rat vascular smooth muscle cells [50]. Both normal and cancerous human cell lines have also been tested in these studies. Normal cell lines that were investigated included mononuclear cells [56], embryonic skin fibroblasts [49], keratinocytes [57], and foreskin fibroblasts [51]. A cancer cell line, i.e., human hepatoma cells, was also examined [29,46,47]. Hydrogen peroxide (H_2_O_2_), 2,2′-azobis(2-amidinopropane) dihydrochloride (AAPH), and ultraviolet (UV) radiation have been commonly used to generate oxidative-stressed cellular models for the characterization of tilapia-derived peptides. H_2_O_2_ is a ROS that can be converted to highly reactive hydroxyl radicals [58]. AAPH is an azo compound that generates free radicals in the cells [59]. UV radiation could lead to the generation of ROS and RNS in cells and tissues [60].

Protein hydrolysates prepared from different tilapia tissues have been shown to protect against oxidative cellular injury (Table 4). The hydrolysates could generally reduce intracellular ROS production in a dose-dependent manner [47,51,55,56]. Moreover, tilapia-derived hydrolysates effectively reduced superoxide production and lactate dehydrogenase (LDH) release from the oxidative-stressed cells [50,55]. The reduction in LDH release is an indicator of lesser oxidative cellular membrane injury [61]. The hydrolysates can also restore cellular enzymatic and non-enzymatic antioxidant defenses, such as superoxide dismutase (SOD) activity and reduced glutathione (GSH) content, in cells undergoing oxidative stress [51,55].

In addition to dampening ROS production, tilapia hydrolysate protected skin fibroblasts against UV-induced oxidative damage by reducing the secretion of matrix metalloproteinases (MMPs), including MMP-1, MMP-3, and MMP-12 [51]. Suppression of MMP secretion is important to the maintenance of skin function as MMPs can degrade extracellular matrix proteins such as collagen and elastin, ultimately reducing skin elasticity [62]. Other than MMP inhibition, tilapia hydrolysates were found to increase the secretion of type 1 procollagen, collagen I, and elastin, which can enhance wound healing and promote skin health [49,51].

Oral administration of protein hydrolysates may modify their bioactivities due to hydrolysis by GI proteases. In light of this, some studies have investigated the effect of simulated GI digestion on the antioxidant potency of tilapia hydrolysates. Zhang et al. [47] reported that following pepsin–pancreatin hydrolysis, the ability of tilapia hydrolysate to dampen ROS production in human hepatocellular carcinoma (HepG2) cells was enhanced. This improvement may be attributed to the GI digesta being more readily absorbed by the cells compared to pre-GI-digested hydrolysate. It is of great interest to investigate whether in vivo GI digestion of a tilapia hydrolysate in experimental rodents and the human body could also enhance the in vivo antioxidant effects of the hydrolysate in a similar manner.

Tilapia peptide–oligosaccharide complexes have been reported to show stronger cellular antioxidant activities than their individual components [63]. These complexes increased the activities of endogenous enzymatic antioxidants, such as SOD and glutathione peroxidase (GSH-Px), in UV-irradiated mouse embryonic fibroblasts and human epidermal keratinocyte (HaCaT) cells. Furthermore, the level of malondialdehyde (MDA) in these photoaged cell models was significantly reduced. MDA is one of the final products of lipid peroxidation and is commonly used as a biomarker of oxidative stress in cellular and animal models [1].

The antioxidant properties of red tilapia viscera hydrolysate (RTVH) were found to be associated with the antiproliferative capacity in three cancer cell lines: HepG2, hepatocyte-derived carcinoma (Huh7) cells, and human colon adenocarcinoma (SW480) cells [64]. Notably, RTVH displayed selective cytotoxicity against SW480 cells, without compromising normal human colon (CCD-18Co) cells. It has been suggested that RTVH causes membrane rupture in cancer cells without inducing apoptosis. However, further investigation has to be conducted to identify the exact mechanism of this selective cytotoxicity, as another study found that the viability of oxidative-stressed cancer cells was not exacerbated by tilapia-derived protein hydrolysates [47].

**Figure 2:**
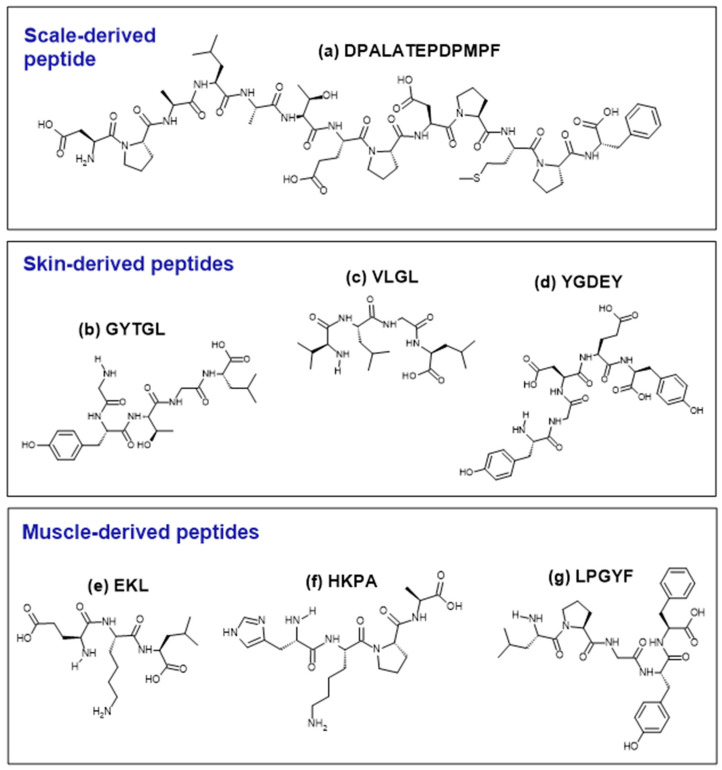
Structures of selected examples of antioxidant peptides derived from the tilapia scale (**a**), skin (**b**–**d**), and muscle (**e**–**g**). Peptides were drawn using the ACD/ChemSketch freeware (version 2022.1.0, Advanced Chemistry Development, Inc. (ACD/Labs), Toronto, ON, Canada, www.acdlabs.com).

Table 5 summarizes the discovery of 16 antioxidant peptides from tilapia protein hydrolysates. Figure 2 shows the structures of selected examples of antioxidant peptides identified from different tilapia tissues. These peptides exhibited multiple beneficial actions beyond attenuating ROS production in oxidative-stressed cells. For example, DPALATEPDPMPF, identified from tilapia scale, and YGDEY, identified from tilapia skin, reduced DNA oxidative damage in murine and human cells, respectively [43,53,54,57]. YGDEY was also shown to enhance the antioxidant defense system in human cell models by increasing SOD activity and GSH levels [46,47,57]. Other than serving as an antioxidant, YGDEY was found to induce cell growth, as reflected by the higher viability of peptide-treated cells compared to non-treated cells [42,57]. The precise mechanism by which YGDEY enhances cell viability is yet to be investigated.

Zhang et al. [29] investigated the antioxidant activity of nine tilapia peptides and of six fragments predicted from those peptides through in silico GI digestion (Table 5). All 15 peptides exhibited ROS scavenging activity in AAPH-treated HepG2 cells. Notably, at 50 μM, four peptides, SC, CH, PGY, and LPGYF, scavenged ROS more effectively than ascorbic acid. SC, CH, and PGY also upregulated the expression of genes encoding catalase (CAT) and SOD in the oxidative-stressed cells. By contrast, *GPx1* gene expression was suppressed by the three peptides, suggesting a not-so-important role of GPx-mediated H_2_O_2_ degradation in the action of the three peptides. The study reported that the outcome of a non-cell-based antioxidant assay, such as an ABTS scavenging assay, may not be able to predict accurately the outcome of a cellular antioxidant assay. Thus, while non-cell-based assays can provide initial insights, the importance of cell-based assays in assessing the antioxidant activity of peptides in a biologically relevant context cannot be overemphasized. Taken together, the study attributed the antioxidant capacity of the peptides to direct intracellular ROS scavenging activity and stimulation of cellular antioxidant enzyme activity [29].

Current research has shed insights into the potential of four tilapia skin-derived antioxidant peptides for cosmetic applications. Three peptides, GYTGL, LGATGL, and VLGL, were shown to prevent and regulate photoaging in ultraviolet B (UVB)-irradiated mouse embryonic fibroblast cells [42]. These peptides significantly inhibited intercellular MMP-1 activity and ROS production in the UVB-irradiated fibroblast cells. Hydrogen bonds and C terminate GL were proposed to play an important role in increasing collagen production in these cells [42]. Another tilapia skin-derived peptide, YGDEY, was found to protect against UVB-induced photoaging in HaCaT cells [57]. YGDEY significantly decreased intracellular ROS levels and increased the expression of endogenous antioxidants such as SOD and GSH in the HaCaT cells. The peptide also protected the DNA of HaCaT cells from UV-induced oxidative damage. Downregulation of the mitogen-activated protein kinase and nuclear factor-kappa B signaling pathway, which suppresses inflammation, was proposed to underlie the inhibition of MMP-1 and MMP-9 expression in the HaCaT cells [57]. The protection against photoaging-induced cellular damage is not limited to tilapia-derived antioxidant peptides. Similar protective effects have been observed with antioxidant peptides derived from the cardiac arterial bulbs of skipjack tuna [65], oysters [66], and the marine microalgae *Isochrysis zhanjiangensis* [67]. Collectively, the promising results obtained in the aforementioned studies on GYTGL, LGATGL, VLGL, and YGDEY strongly warrant future investigation in animal models to evaluate their ability to prevent UV-induced skin aging.

In recent years, molecular docking has emerged as a crucial technology in computer-aided drug research. This technique could be employed to predict the preferred orientation of a peptide when it binds to a target protein, which is essential for understanding the molecular interactions that drive the bioactivity of the peptide [68]. Through molecular docking simulation, Xiao et al. [57] predicted that YGDEY can form seven hydrogen bonds with MMP-1 and MMP-9, consequently stabilizing the protein–peptide complexes and inactivating the MMPs. Similarly, molecular docking analysis suggests that the binding of GYTGL, LGATGL, and VLGL to the active site of MMP-1 potentially contributes to MMP-1 inhibition in photoaged cells [42]. On the other hand, molecular docking analysis also revealed a strong affinity of YGDEY towards bcl-2, an anti-apoptotic protein, facilitated by hydrogen bonding. This observation is in line with increased bcl-2 expression in ethanol-treated HepG2 cells, and potentially improved cell viability [46].

Owing to the various assays and conditions employed to characterize the antioxidant potency of tilapia-derived hydrolysates and peptides, comparison between studies is not always straightforward. For example, when characterizing the cellular antioxidant activity of tilapia peptide or hydrolysate, Kangsanant et al. [48] measured the level of NO, whereas Sierra et al. [50] measured the level of O_2_^●−^. Multiple types of free radicals may be targeted by tilapia antioxidant peptides and hydrolysates. Thus, in future studies, a more comprehensive approach that characterizes the action of tilapia peptides or hydrolysates against both ROS and RNS would provide a more conclusive understanding of their antioxidant effects.

## 5. In Vivo Effects

In vivo assays are indispensable in demonstrating the physiological relevance and efficacy of tilapia-derived antioxidant peptides and protein hydrolysates in living organisms. A whole organism model allows investigation of the impact of peptide administration on oxidative stress biomarkers, tissue damage, and disease progression in vivo. At present, the number of in vivo studies on the antioxidant activities of tilapia peptide mixtures and tilapia protein hydrolysates is still limited, as summarized in Table 6. The studies employed rodents as experimental models for diseases or pathological conditions such as kidney and liver injury in aging [23], diabetes [22,69], hypertension [44], and skin photoaging [24,70,71]. All the studies reported positive results in alleviating the symptoms or damages in the disease models. However, none of the studies tested individual tilapia-derived peptides on the animal models; all of them focused on peptide mixtures or protein hydrolysates. Sun et al. [70] identified the peptide sequence LSGYGP, which exhibited in vitro antioxidant activity, from tilapia gelatin hydrolysate. Ren et al. [72] identified the peptide sequences GO, ED, DOG, EPPF, and KPFGSGAT from an antioxidant fraction of tilapia skin collagen hydrolysate. Meanwhile, a tilapia collagen polypeptide sample shown to be effective in alleviating oxidative injury in aging mice was found to consist of 41 peptide chains, ranging from 7 to 22 amino acids [23]. These findings imply that perhaps some or all of such peptides might have contributed to the in vivo antioxidant activity of the tilapia hydrolysates that they were identified from. The in vivo antioxidant effect of each of those peptides and how they interact to contribute to the overall antioxidant activity observed in a hydrolysate remain unknown.

In vivo antioxidant effects of tilapia peptide mixtures and hydrolysates were typically assessed via biomarkers such as antioxidant enzyme activities (e.g., SOD, CAT, GSH, and GSH-Px) [23,24], in addition to oxidative stress biomarkers (e.g., ROS and MDA) [24,44] (Table 6). Notably, some studies demonstrate the multifunctionality of tilapia peptide mixtures and hydrolysates in vivo. For instance, tilapia collagen peptide mixture TY001, a patented peptide mixture containing tilapia collagen peptides as the major component, exerts antioxidant, anti-diabetic, wound-healing, and anti-inflammatory effects in vivo [69]. TY001 promotes wound healing in diabetic mice by restoring SOD and CAT activities, enhancing collagen deposition and hydroxyproline levels in the wound tissues, as well as modulating in vivo inflammatory and anti-inflammatory cytokine production [69]. In vivo antioxidant and anti-inflammatory effects were also found for a commercial tilapia collagen peptide powder containing peptides of 500–3000 Da in size [24]. Furthermore, a tilapia skin collagen hydrolysate enriched in peptides of <1000 Da (about 77%) was reported to have both antioxidant and anti-diabetic effects in vivo [22].

Although some studies did not directly measure in vivo antioxidant or oxidative parameters in their experiments, they have demonstrated the protective effects of tilapia hydrolysates and peptide mixtures in their animal studies. For instance, Gao et al. [73] found that the oral intake of a commercial tilapia skin hydrolysate mitigated colitis symptoms and colonic damage induced by dextran sulfate sodium (DSS) in mice. On the other hand, Xiong et al. [74] demonstrated the anti-inflammatory effect of TY001 in a wounded zebrafish model. It should be noted that an anti-inflammatory agent is not necessarily an antioxidant agent, although some tilapia peptides do possess both antioxidant and anti-inflammatory activities in vivo (Table 6). Therefore, future investigation is warranted to determine whether tilapia peptides also exhibit in vivo antioxidant effects in the aforementioned DSS-induced colitis mouse and wounded zebrafish models. Meanwhile, Lu et al. [75] reported the in vivo protective effects of a hydrogel containing tilapia skin collagen polypeptides (<5 kDa) against alcohol-induced liver and brain injury in mice. Although the in vitro antioxidant potency of the hydrogel was demonstrated, its in vivo antioxidant effect was not investigated. This question is of interest as it is unclear whether the tilapia peptide-incorporated hydrogel still retains its antioxidant activity following oral administration into the animal model and in vivo digestion.

Exposure to UV light triggers significant ROS production and DNA damage in skin tissue, leading to oxidative stress. UV exposure induces maximal ROS and MDA levels, underscoring the severity of oxidative stress in skin tissues [76]. Two studies reported the protective effects of tilapia-derived peptide samples against UV-induced skin photoaging in mice [24,70]. Song et al. [24] reported that tilapia collagen peptides per se were able to enhance antioxidant enzyme activities, dampen ROS production, and reduce the levels of inflammatory factors. Nevertheless, their impact on restoring skin integrity was insignificant. A much stronger protective effect on skin barrier integrity was detected when tilapia collagen peptides were administered to the mice in combination with natural antioxidant supplements. Oral administration of tilapia collagen peptides alone or in combination with natural antioxidant supplements was also found to increase the mRNA expression of the *nuclear factor erythroid 2-related factor 2* gene; the gene codes for a master transcription factor that modulates various antioxidant genes in cells [24]. Sun et al. [70] found that the anti-photoaging effects of tilapia gelatin hydrolysate lies mainly in their antioxidant effects and their protection against UV-induced collagen damage. Thus, both studies pointed to the potential application of tilapia-derived peptides in the formulation of functional food ingredients or dietary supplements for protection against UV-induced oxidative stress and skin aging [24,70].

While the studies in Table 6 mostly used aging or disease-induced rodent models to examine the antioxidant effects of tilapia peptide mixtures and hydrolysates, the benefits of tilapia peptides have also been examined in a healthy rodent model subjected to physiological stress, i.e., exhaustive exercise. Ren et al. [72] reported antifatigue effects of a tilapia skin collagen hydrolysate on mice subjected to exhaustive swimming, which can induce oxygen deficit, prompting the generation of free radicals that disrupt muscle tissues. The authors proposed that enhancement of endogenous antioxidant enzymes, including SOD, could be one of the mechanisms through which tilapia hydrolysate can protect against exercise-induced oxidative injury and fatigue.

## 6. Molecular Characteristics and Structure–Activity Relationship

The antioxidant activity of a peptide is closely related to its molecular size, amino acid composition, and sequence [1,7]. Antioxidant peptides that contain fewer than 20 amino acid residues can cross the intestinal barrier to exert their biological effects [77]. Furthermore, smaller peptides can effectively penetrate through cell membranes [78,79], contributing to high intracellular ROS scavenging capacity. Thus, unsurprisingly, all tilapia-derived antioxidant peptides presented in Table 7 are shorter than 20 amino acids, except for the two peptides reported by Kangsanant et al. [48]. Typically, food-derived antioxidant peptides range between 500 and 1800 Da in molecular weight [1,3,7]. In agreement with this, 14 of the 16 tilapia-derived antioxidant peptides have molecular weights ranging between 372 and 1383 Da (Table 7).

Kangsanant et al. [48] identified two relatively large antioxidant peptides of >6000 Da from tilapia muscles (Table 7). Their finding suggests that peptide size is clearly not the sole determinant of ROS scavenging capacity, at least not in the two large peptides. However, it is important to note that peptides of high molecular weight may encounter challenges in crossing the intestinal barrier intact, impairing GI absorption [81]. Additionally, large peptides may also be more susceptible to proteolysis by digestive enzymes in the GI tract, potentially losing the initial amino acid sequence or composition that accounts for their original antioxidant potency. Thus, high-molecular-weight peptides that exhibit antioxidant effects in cell models may not exert similar effects in animal models.

The presence of hydrophobic amino acids is known to contribute to the antioxidant activities of peptides [1,82]. Ranathunga et al. [83] reported that hydrophobic amino acid residues could increase the presence of peptides at the water–lipid interface, facilitating their access to scavenge free radicals generated at the lipid phase. Hydrophobic amino acids may make peptides easier to dissolve in lipid, contributing to the peptides’ cellular antioxidative effect [84]. In line with this, tilapia-derived antioxidant peptides generally contain 20–100% hydrophobic residues in their sequences (Table 7). Interestingly, the two atypically large antioxidant peptides identified by Kangsanant et al. [48] also had a high proportion of hydrophobic amino acids, exceeding 50% of their total composition (Table 7). The major amino acid residues of the two peptides were Ala, Asp, Lys, Gly, and Phe. The presence of alkyl, amino, or carboxylic groups in the side chains of these amino acids may enable them to act as hydrogen donors when scavenging free radicals. Due to their large size, the two peptides likely exerted their antioxidant activity outside cell membranes, lowering intercellular free radical concentration [48].

Other than hydrophobic amino acids, the aromatic amino acid Tyr can also act as a hydrogen donor, which delivers a proton to suppress free radical generation [85]. Despite being hydrophilic, His-containing peptides can exert antioxidant effects through the hydrogen-donating, lipid peroxyl radical-trapping, and metal ion-chelating actions of the His imidazole group [86]. Thus, the presence of His residues may at least in part explain the ROS scavenging activities of HKPA, ASLCH, and SLCH [29].

The intracellular ROS scavenging ability of peptides might also be influenced by the amino acid residues at the terminal ends [1]. Examination of the 16 tilapia-derived antioxidant peptides in Table 7 revealed 12 peptides containing a hydrophobic amino acid residue at the N- or C-terminus, or both termini (EKP, EKL, ALSC, HKPA, ASLCH, LPGYF, LEVPGY, VLGL, GYTGL, LGATGL, DPALATEPDPMPF, and AFAVIDQDKSGFIEEDELKLFLQNFSAGARAGDSDGDG-KIGVDEFAALVK). Despite the absence of hydrophobic amino acid at the terminal ends, YGDEY contains the aromatic residue Tyr at both N- and C-termini, in addition to having a Gly residue adjacent to the N-terminus [57]. The antioxidant property of the Tyr residue can be attributed to its ability to act as a hydrogen donor and its strong affinity towards hydrophobic proteins. The Gly residue near the N-terminal can also act as a hydrogen donor when combined with unpaired electrons and radicals. On the other hand, the C-termini of GYTGL, LGATGL, and VLGL comprise the GL sequence, which was proposed by Liping et al. [42] to be crucial for the radical scavenging ability of the three peptides.

For antioxidant peptides, important contributing factors include high proportions of hydrophobic amino acids, as well as the presence of selected amino acids (Tyr, Met) [27]. In a comparative study on fish-derived antioxidant peptides covering 12 fish species (ranging from freshwater to seawater fishes, and from tropical to temperate species), tilapia-derived antioxidant peptide was found to be the only fish peptide containing the amino acid Met [87]. Similarly, tilapia antioxidant peptides can contain up to 100% hydrophobic amino acid residue (Table 7), compared to 76% in mudskipper-derived antioxidant peptides (tripeptides and above) [88]. Both the presence of Met and the high hydrophobicity contribute to the tilapia peptides’ superior antioxidant potential.

## 7. Potential Applications in Human Health

The discovery of antioxidant protein hydrolysates and peptides from tilapia opens up possibilities for their potential application in human health. As highlighted above, tilapia-derived hydrolysates and peptides can exert antioxidant activity and other bioactivities in various biological models (see Table 4, Table 5 and Table 6). Thus, they have the potential to be developed into various food, cosmeceutical, and therapeutic products that can protect the human body against oxidative stress.

Tilapia-derived hydrolysates and peptides can be developed into multifunctional dietary supplements or incorporated into functional foods to boost the natural antioxidant defenses in the body. Such supplements or functional foods can be used to target consumers who are at risk of oxidative stress, such as those with chronic diseases and those at risk of oxidative stress due to unhealthy diets, exposure to environmental pollutants, and/or lack of exercise [89]. The use of protein hydrolysates in the development of supplements or functional food ingredients may be more economical compared to using pure peptides [32]. For example, tilapia collagen peptide mixture TY001, which exhibited antioxidant, anti-inflammatory, and wound-healing effects in diabetic mice [69], could be developed into multifunctional dietary supplements and functional foods for people with diabetes. Similarly, the RTVH that showed antiproliferative effects against cancer cells [64] could also be developed into products conferring protection against cellular or tissue oxidative injury and cancer risks. On the other hand, the peptide DPALATEPDPMPF, which exhibits antihypertensive and antioxidant activities, is a promising candidate as a functional ingredient for the development of health food addressing hypertension and oxidative stress [54]. KPFGSGAT, a peptide identified from tilapia skin collagen hydrolysate and exhibiting antioxidant and antifatigue effects in mice, is also a promising candidate for the development of functional food ingredients [72].

Antioxidant peptides and hydrolysates derived from tilapia can also be used to formulate cosmetic products to protect the skin from UV-induced damage. Examples of promising anti-photoaging candidates that can be explored for the development of cosmetics and oral cosmeceuticals include (i) tilapia scale-derived hydrolysate, which protects human fibroblasts against ultraviolet A radiation [51], (ii) peptide YGDEY, which protects human keratinocytes against UVB radiation [57], and (iii) LSGYGP, a peptide identified from tilapia gelatin peptides that protects against UV-induced photoaging in mice [70].

The protective effects of tilapia-derived antioxidant hydrolysates and peptides have been demonstrated in various disease models, suggesting their potential application as therapeutic agents for the treatment of oxidative stress-mediated pathological conditions. The hydrolysates and peptides may serve as leads for a more thorough discovery of peptide-based drugs, subjected to further modification or optimization to enhance their potency to that expected of therapeutic agents. For instance, the RTVH with antiproliferative effects against cancer cells [64] can be further explored to identify specific constituent peptides with potential anticancer effects. Meanwhile, YGDEY, which suppresses the inflammatory process in human cells [57], is also a promising candidate for future anti-inflammatory drug discovery.

## 8. Future Perspectives

While understanding about the mechanisms by which they exert antioxidant effects in cells and animal models is expanding, further research is still needed to realize the human health benefits of tilapia peptides and hydrolysates. Future work is required to elucidate the antioxidant effects of tilapia peptides at the cellular and molecular levels more comprehensively. Most of the studies reviewed here have focused on the effects of tilapia peptides on the activities of antioxidant enzymes that protect against cellular and tissue oxidative damage [24,43,46,51,69]. However, these studies have largely overlooked the potential role of pro-oxidative enzymes, which can contribute to cellular ROS production. Future studies should also investigate the possibility of tilapia peptides and hydrolysates modulating the activities of pro-oxidative enzymes, such as lipoxygenase, myeloperoxidase, nicotinamide adenine dinucleotide phosphate oxidase, and xanthine oxidase. Dysregulation of such enzymes leads to cellular oxidative damage; their potential roles as therapeutic targets have also been highlighted in recent reviews [90,91,92,93]. Future research should also explore more thoroughly the molecular mechanisms by which tilapia-derived peptides exert their antioxidant effects in cells and in vivo, especially the regulation of genes and signaling pathways involved in modulating cellular redox balance.

Further animal studies are needed to validate the efficacy of tilapia peptides in a broader range of models beyond those discussed above. Expanding these studies to additional animal models may uncover new health benefits of the peptides. One promising direction is to investigate the impact of tilapia antioxidant peptides on animal models of brain aging and neurodegenerative diseases, which are conditions associated with excessive free radical production [94]. These studies may prioritize tilapia-derived peptides whose cellular antioxidant effects were well documented but whose in vivo effects are unknown. Examples of such peptides include DPALATEPDPMPF, GYTGL, LGATGL, VLGL, and YGDEY, as shown in Table 5. Such studies would allow the in vivo bioavailability and potency of such peptides to be examined, paving the way for their potential development as functional food ingredients or therapeutic agents in the future. In particular, the resistance of tilapia antioxidant peptides and hydrolysates to degradation in the GI tract and bloodstream can be investigated more effectively in in vivo models than in cellular models. To date, there are no reports on the toxicity of tilapia-derived antioxidant peptides and hydrolysates. However, potentially toxic and allergenic peptides may be released during enzyme hydrolysis [95]. Therefore, tilapia-derived antioxidant peptides and hydrolysates should be tested for in vivo toxicity and allergenicity before their future application in food and human health. Eventually, rigorous clinical trials are needed to assess the safety, bioavailability, and pharmacokinetic profiles of tilapia-derived antioxidant peptides and hydrolysates, which will set the stage for their potential applications in human health.

Rodent models have been used to demonstrate the in vivo antioxidant effects of tilapia-derived peptide mixtures and hydrolysates, as discussed above. While mammalian models are genetically more relevant for mimicking human disease models, other in vivo models, such as *Caenorhabditis elegans*, can be considered in the future high-throughput discovery of antioxidant peptides from tilapia. *C. elegans* has numerous advantages as an in vivo model organism, such as its transparent body, short lifespan, and ease of maintenance, and the availability of well-established experimental methodologies [96]. To date, *C. elegans* has been successfully used as a model for evaluating food-derived antioxidant peptides [97,98,99]; thus, future research may consider using *C. elegans* as an in vivo model to expedite the discovery of antioxidant peptides from tilapia.

The conventional strategy used for the isolation of tilapia antioxidant peptides with cellular and in vivo antioxidant activities can be further improved. Firstly, the range of proteases used for peptide production from tilapia proteins can be expanded, considering that there are more than 40 proteases of plant, animal, and bacterial origins that are commercially available [100]. By exploring different enzymes, enzyme combinations, and hydrolysis conditions, it may be possible to optimize the yield of antioxidant peptides from tilapia, in addition to generating novel peptides with improved cellular and in vivo antioxidant activities. Secondly, techniques that have been shown to improve the isolation of bioactive peptides from other protein sources can be applied to improve the isolation of antioxidant peptides from tilapia. Two examples of such techniques are pulsed electric field (PEF) and electro-membrane filtration (EMF). PEF treatment prior to enzymatic hydrolysis has been reported to facilitate enzyme access to microalgae proteins [101]. Meanwhile, EMF, which combines the separation mechanisms of membrane filtration and electrophoresis, has been used to isolate bioactive peptides from an alpha(s2)–casein hydrolysate. EMF has a greater selectivity and cost-effectiveness compared with conventional isolation techniques such as membrane filtration and chromatography [102]. Thirdly, future studies may also incorporate free online tools into their wet-lab experimentations to accelerate the screening and discovery of peptides, which can be further validated in cellular and animal models. Two examples of such tools are the BIOPEP-UWM database [103] and AnOxPePred [104], which can be used to perform in silico proteolysis to screen for potential enzyme treatments for hydrolysate production and to predict potential antioxidant peptides.

Tilapia-derived peptides and hydrolysates that exhibit antioxidant activity in biological models are promising candidates for application in functional food formulation. In this context, the stability and bioactivity of these peptides in different food matrices should be explored in the future. Compatible food products to serve as carriers for tilapia antioxidant peptides should be those in which the peptides are well protected from degradation during food processing and storage and until consumption [105]. If the incorporated peptides can protect against food protein and lipid oxidation, they may also contribute to food quality preservation. Furthermore, an interesting question for future research is the possibility of synergism between tilapia peptides and other antioxidants present in food matrices, and how this may impact food quality preservation and the in vivo benefits of food products when consumed.

Bitterness and fishy odor are challenges that need to be addressed when incorporating tilapia-derived peptides and hydrolysates into food products. The bitterness of peptides is a challenge faced by the food protein hydrolysate industries, as consumers may be unwilling to accept a bitter taste for health benefits [106,107]. Meanwhile, lipid oxidation may contribute to fishy odor development in tilapia protein hydrolysates [108]. Some strategies are known to reduce the bitterness and fishy odor of peptides and protein hydrolysates, such as the application of multi-step enzymatic hydrolysis [109], masking agents, flavoring agents, and spray-drying microencapsulation [110,111]. The applicability of such strategies in the formulation of functional foods incorporating tilapia antioxidant peptides and hydrolysates should be investigated in the future.

Lastly, comparisons of antioxidant peptides derived from tilapia cultured under different farming practices and types of fish feed are limited. Fish farming practices and feed sources have been reported to influence the protein contents of Nile tilapia [112]. In addition, the quality of tilapia antioxidant peptides may also be influenced by the quality or source of tilapia used for peptide and hydrolysate production [113]. Thus, future research is warranted to investigate the impact of tilapia sources and farming conditions on the antioxidant functionality of derived peptides. Comparing peptides derived from tilapia cultured under different systems could provide insight into optimal farming strategies for producing tilapia as a source of high-quality antioxidant peptides and hydrolysate. To this end, industry–academia collaboration is important for realizing the potential of tilapia as a sustainable source of antioxidant peptides for functional food and nutraceutical applications in the long term.

## 9. Conclusions

Current cellular and in vivo evidences have established the antioxidant capacity of tilapia-derived protein hydrolysates and peptides. To date, only a small number of antioxidant peptide sequences have been identified from tilapia, whose cellular antioxidant actions have been demonstrated. Meanwhile, in vivo evidence was largely derived from investigations on tilapia protein hydrolysates, not individual peptide sequences. Generally, tilapia-derived peptides and hydrolysate were shown to enhance cellular and tissue antioxidant defense and attenuate oxidative injury. Additionally, they exerted additional functions relevant to human diseases and pathological conditions associated with oxidative stress, as exemplified by their wound-healing, antiaging, anti-diabetic, and anti-inflammatory properties in vivo. Together, such evidence highlights the potential applications of tilapia-derived peptides and hydrolysates in functional foods, health supplements, and therapeutic agents for human health. Currently, the in vivo bioavailability, efficacy, and safety of the reported peptides and protein hydrolysates remain not well established. Much future research on tilapia-derived antioxidant peptides and protein hydrolysate is still required to realize their potential applications in human health.

## Figures and Tables

**Table 1:** Global tilapia production (thousand tons, live weight) by aquaculture type and year.

Aquaculture Type	Tilapia Species	Year				
		2000	2005	2010	2015	2020
Inland Aquaculture	Nile tilapia (*Oreochromis niloticus*)	1001.5	1721.3	2637.4	4000.9	4407.2
	Tilapias nei (*Oreochromis* spp.)	123.9	199.3	449.6	929.9	1069.9
Marine and Coastal Aquaculture	Nile tilapia (*Oreochromis niloticus*)	1.6	5.3	20.3	49.8	107.4

(Source: Hush [37]).

**Table 2:** Examples of enzymatic hydrolysis conditions used to produce antioxidant protein hydrolysates from various tilapia tissue samples ^a^.

Tilapia Part	Enzyme Used for Hydrolysis	Hydrolysis Condition (pH; Temperature; Duration)	DH (%)	Yield (%)	Reference
Muscle	Alcalase^®^	pH 8; 50 °C; 10 h	35.0	12.4	[29]
Muscle	Flavourzyme^®^	pH 7; 55 °C; 1 h	13.3	-	[48]
Dorsal meat	Alcalase^®^ followed by pepsin and pancreatin	Alcalase^®^: pH 8; 50 °C; 2, 6, 10 and 16 h Pepsin: pH 3; 37 °C; 2 h Pancreatin: pH 7; 37 °C; 2 h	40–55	-	[47]
Skin	Alcalase^®^ followed by Pronase E^®^	Alcalase^®^: pH 8; 50 °C; 30 min Pronase E^®^: pH 8; 50 °C; 2 h	-	-	[49]
Skin	Pepsin followed by pancreatin	Pepsin: pH 2.5; 37 °C; 1 h Pancreatin: pH 7.5; 37 °C; 2 h	-	-	[42]
Skin	Multifect neutral^®^ followed by Properase E^®^	Multifect neutral^®^: pH 8.0; 35 °C; 4.5 h Properase E^®^: pH 9.0; 55 °C; 4.5 h	22.1	-	[43,46]
Skin	Neutral protease and papain	pH 7; 50 °C; 3 h	-	-	[23]
Scales	Alcalase^®^	pH 8; 58.5 °C; 55 min	12	86	[50]
Scales	Trypsin	pH 7; 55 °C; 4 h	-	-	[51,52]
Viscera	Alcalase^®^	pH 8; 55 °C; 1.5 h	41.5	-	[44,45]

^a^ Information not reported. DH, degree of hydrolysis.

**Table 3:** Examples of purification and identification methodologies used in the discovery of antioxidant peptides from tilapia ^a^.

Purification Strategy	Peptide Identification	Peptide Sequence	Reference
GFC, RP-HPLC	MS/MS	GYTGL LGATGL VLGL	[42]
GFC, RP-HPLC	LC-MS/MS	KAFAVIDQDKSGFIEEDELKLFLQNFSAGARAGDSDGDGKIGVDEFAALVK AFAVIDQDKSGFIEEDELKLFLQNFSAGARAGDSDGDGKIGVDEFAALVK	[48]
GFC, RPC	LC–MS/MS	EKL EKP HKPA ELSC ALSC ASLCH SLCH LPGYF LEVPGY	[29]
AEC, RP-HPLC	MS/MS	DPALATEPDPMPF	[53,54]
GFC, CEC, RP-HPLC	MS/MS	YGDEY	[43,46]

^a^ AEC, anion exchange chromatography; CEC, cation exchange chromatography; GFC, gel filtration chromatography; LC-MS/MS, liquid chromatography coupled with MS/MS; MS/MS, tandem mass spectrometry; RP-HPLC, reversed-phase high-performance liquid chromatography; RPC, reversed-phase chromatography.

**Table 4:** The protective effects of protein hydrolysates from different tilapia tissues in cells under oxidative stress ^a^.

Part of Tilapia	Species	Protease Used for Hydrolysis	Sample Dose	Cell Model	Key Findings	Reference
Muscle	*Oreochromis niloticus*	Protease A “Amano” 2 Protease N “Amano” Cryotin-F Flavourzyme^®^ Neutrase^®^	7.5–25%	H_2_O_2_-treated mononuclear cells Phorbol myristate acetate-treated mononuclear cells	Increased radical scavenging ability Reduced ROS production	[56]
Skin	*Oreochromis niloticus*	Alcalase^®^ followed with Pronase E^®^	1.56–12.5 mg/mL	AAPH-treated Detroit 551 cells	Increased cell viability against oxidative damage	[49]
Skin	*Oreochromis niloticus*	Ginger protease	0.5–10 mg/mL	H_2_O_2_-treated IPEC-J2 cells	Reduced ROS production Reduced LDH release Increased transepithelial electrical resistance Increased expression of γ-glutamylcysteine ligase and GSH level	[55]
Scales	*Oreochromis* sp.	Alcalase^®^	2 mg/mL	Angiotensin II-treated A7r5 cells	Reduced O_2_^●−^ production	[50]
Scales	*Oreochromis niloticus*	Trypsin	50–500 µg/mL	UVA radiation-treated HFF-1 cells	Reduced ROS production Increased SOD activity and GSH level Increased cell migration Reduced MMP-1, MMP-3, and MMP-12 secretion Increased collagen I and elastin secretion	[51]
Dorsal meat	*Oreochromis niloticus*	Alcalase^®^ followed with pepsin and pancreatin	0.1–5.0 mg/mL	AAPH-treated HepG2 cells	Reduced ROS production	[47]

^a^ AAPH, 2,2′-azobis(2-amidinopropane) dihydrochloride; A7r5, rat vascular smooth muscle; Detroit 551, human embryonic skin fibroblast; GSH, reduced glutathione; HepG2, human hepatocellular carcinoma; HFF-1, human foreskin fibroblast; IPEC-J2, porcine jejunal; LDH, lactate dehydrogenase; MMP, matrix metalloproteinase; O_2_^●−^, superoxide; ROS, reactive oxygen species; SOD, superoxide dismutase; UVA, ultraviolet A.

**Table 5:** Cellular effects of peptides derived from *O. niloticus* protein hydrolysates ^a^.

Part of Tilapia Hydrolyzed	Protease Used for Hydrolysis	Peptide Identified	Sample Dose	Cell Model	Key Findings	Reference
Scale	Alcalase^®^	DPALATEPDPMPF	20–200 µg/mL [53] 1–20 µg/mL [54]	H_2_O_2_-treated RAW 264.7 cells H_2_O_2_-treated BV-2 cells	Reduced ROS production Reduced DNA oxidative damage	[53,54]
Muscle	Flavourzyme^®^	KAFAVIDQDKSGFIEEDELKLFLQNFSAGARAGDSDGDGKIGVDEFAALVK	100 µg/mL	H_2_O_2_-treated RAW 264.7 cells	Reduced ROS production	[48]
		AFAVIDQDKSGFIEEDELKLFLQNFSAGARAGDSDGDGKIGVDEFAALVK	100 µg/mL	Lipopolysaccharide-treated RAW 264.7 cells	Reduced NO production	
Skin	Pepsin followed with pancreatin	GYTGL LGATGL VLGL	50–200 µg/mL	UVB radiation-treated MEF cells	Reduced ROS production Reduced MMP-1 activity Increased collagen production	[42,43]
Skin	Multifect neutral^®^ followed with Properase E^®^	YGDEY	10–100 µM	UVB radiation-treated HaCaT cells	Reduced ROS production Reduced DNA oxidative damage Increased SOD activity and GSH level Reduced MMP-1 and MMP-9 secretion/activity Increased procollagen I production Suppressed inflammation	[43,57]
			10–100 µM	Ethanol-treated HepG2 cells	Reduced ROS production Reduced DNA oxidative damage Increased SOD activity and GSH level Reduced GGT level Reduced apoptosis	[43,46]
Muscle	Alcalase^®^	EKL EKP HKPA ELSC ALSC ASLCH SLCH LPGYF LEVPGY	10–100 µM	AAPH-treated HepG2 cells	Reduced ROS production	[29]

^a^ AAPH, 2,2′-azobis(2-amidinopropane) dihydrochloride; BV-2, murine microglial; GGT, gamma-glutamyltransferase; GSH, reduced glutathione; HaCaT, human keratinocyte; HepG2, human hepatoma; MEF, mouse embryonic fibroblast; MMP, matrix metalloproteinase; NO, nitric oxide; O_2_^●−^, superoxide; ROS, reactive oxygen species; RAW 264.7, mouse macrophage; SOD, superoxide dismutase; UVB, ultraviolet B.

**Table 6:** In vivo effects of tilapia-derived peptide mixtures and protein hydrolysates ^a^.

Sample	Effective Dosage (per kg Body Weight)	In Vivo Model	Key Findings	Reference
Collagen polypeptides (<3 kDa) prepared from tilapia skin using neutral protease and papain	500–2000 mg	Mouse model of aging induced by D-galactose	Alleviated reduction of SOD, CAT, and GSH-Px activities in liver and kidney tissues Decreased MDA levels in liver and kidney tissues Decreased serum LPO Suppressed the induction of protein expression of inducible nitric oxide synthase in liver and kidney tissues	[23]
Tilapia gelatin hydrolysate prepared with Properase E^®^	100–200 mg	UV-induced skin photoaging mice	Increased SOD, CAT, and GSH-Px activities Reduced MDA level Increased collagen deposition	[70,71]
Commercial tilapia collagen peptide powder, in combination with antioxidant supplements	Collagen peptide, 1.2 g; vitamin C, 100 mg; vitamin E, 2.66 mg; astaxanthin, 2.5 mg	UV-induced skin photoaging mice	Increased plasma SOD, GSH-Px, and CAT activities Reduced serum ROS and plasma MDA levels Reduced pro-inflammatory factors, IL-1α, IL-6, IL-10, IL-12, and TNF-α, in serum Increased Hyp and type I collagen contents	[24]
Tilapia skin collagen hydrolysate prepared with Alcalase^®^	0.85–1.70 g	Alloxan-induced diabetic mice	Increased pancreatic SOD and CAT, and reduced MDA levels High dose of peptides showed similar hypoglycemic activity as metformin, an anti-diabetic drug	[22]
Tilapia collagen peptide mixture TY001	20–60 g	STZ-induced diabetic mice	Increased SOD and CAT activities of wound tissues Enhanced wound healing Decreased blood glucose level Reduced level of serum pro-inflammatory cytokines IL-1β and IL-8 Increased level of serum anti-inflammatory IL-10 Increased Hyp contents and enhanced collagen deposition	[69]
Tilapia viscera hydrolysate extract prepared with Alcalase^®^	150–300 mg	DOCA-salt-induced hypertensive rats	Reduced MDA level in kidney Alleviated glomerular necrosis in renal tissues	[44]
Tilapia skin collagen hydrolysate prepared with Alcalase^®^	250–2500 mg	Mice subjected to exhaustive swimming assay	Increased serum SOD level Alleviated fatigue	[72]

^a^ CAT, catalase; DOCA, deoxycorticosterone acetate; GSH-Px, glutathione peroxidase; Hyp, hydroxyproline; IL, interleukin; LPO, lipid peroxidation; MDA, malondialdehyde; ROS, reactive oxygen species; SOD, superoxide dismutase; STZ, streptozotocin.

**Table 7:** Molecular size and percentages of hydrophobic residues in 16 tilapia-derived antioxidant peptides, whose cellular antioxidant activities were demonstrated using cellular models.

Peptide	Molecular Mass (Da)	Hydrophobic Amino Acid Residue (%) ^b^	Reference
EKP	372.42 ^a^	33.3	[29]
EKL	388.46 ^a^	33.3	
ALSC	392.48 ^a^	50.0	
ELSC	450.51 ^a^	25.0	
HKPA	451.53 ^a^	50.0	
SLCH	458.54 ^a^	25.0	
ASLCH	529.62 ^a^	40.0	
LPGYF	595.70 ^a^	60.0	
LEVPGY	676.77 ^a^	66.7	
VLGL	401.28	100.0	[42,43]
GYTGL	510.26	60.0	
LGATGL	531.31	83.3	
YGDEY	645.21	20.0	[43,46,57]
DPALATEPDPMPF	1382.57	69.2	[53,54]
AFAVIDQDKSGFIEEDELKLFLQNFSAGARAGDSDGDGKIGVDEFAALVK	6309.46	56.0	[48]
KAFAVIDQDKSGFIEEDELKLFLQNFSAGARAGDSDGDGKIGVDEFAALVK	6334.49	54.9	

^a^ Molecular mass estimated with an online peptide calculator (https://www.bachem.com/knowledge-center/peptide-calculator/, accessed on 10 June 2024); ^b^ percentages of hydrophobic residues were computed manually, based on the classification of A, F, G, I, L, M, P, V, and W as hydrophobic amino acids [80].

## Data Availability

No new data were created or analyzed in this study. Data sharing is not applicable to this article.
